# Endoscopic resection of early esophageal neoplasia in patients with esophageal varices: a systematic review

**DOI:** 10.1055/a-2524-4148

**Published:** 2025-02-24

**Authors:** Charlotte N. Frederiks, Laura S. Boer, Bas Gloudemans, Lorenza Alvarez Herrero, Jacques J.G.H.M. Bergman, Roos E. Pouw, Bas L.A.M. Weusten

**Affiliations:** 1 Department of Gastroenterology and Hepatology, St. Antonius Hospital, Nieuwegein, Netherlands; 2 Department of Gastroenterology and Hepatology, University Medical Center Utrecht, Utrecht University, Utrecht, Netherlands; 3 Department of Gastroenterology and Hepatology, Amsterdam University Medical Centers, Amsterdam Gastroenterology Endocrinology and Metabolism, Cancer Center Amsterdam, Amsterdam, Netherlands

## Abstract

**Background:**

Although endoscopic resection (ER) is recommended as first-choice treatment for early esophageal neoplasia, patients with esophageal varices are considered a high-risk group owing to an increased risk of bleeding. This systematic review aimed to evaluate the effectiveness and safety of ER in this specific patient category.

**Methods:**

We searched for studies reporting on clinical outcomes of ER in the presence of esophageal varices, irrespective of study design or follow-up time. End points included the incidence of prophylactic measures to reduce the risk of variceal hemorrhage, radical and curative resection rates, and adverse events.

**Results:**

After screening 2371 studies, 42 studies (including our own unpublished cohort) with a total of 186 patients were included in this systematic review. Endoscopic band ligation (72/186; 39%) and endoscopic injection sclerotherapy (22/186; 12%) were the prophylactic measures most widely adopted to eradicate varices prior to ER. Other frequently described prophylactic measures included direct varix coagulation during ER (18/186; 10%) and the placement of a transjugular intrahepatic portosystemic shunt prior to ER (9/186; 5%). While the radical and curative resection rates were high (86% and 72%, respectively), the periprocedural and delayed bleeding risks were reported to be relatively low (6% and 3%, respectively). In all studies, no procedure-related mortality was observed.

**Conclusions:**

ER appeared to be a safe and effective treatment option in selected patients with concurrent early esophageal neoplasia and esophageal varices, provided that a tailored approach of adequate prophylactic measures to prevent bleeding is applied.

## Abstract (de)

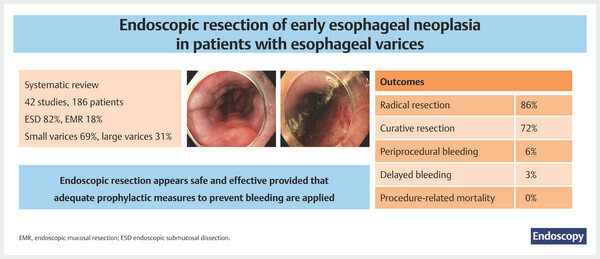

## Introduction

Current guidelines recommend endoscopic resection (ER) as the primary treatment option for early esophageal neoplasia 1 2 3 . Next to the curative potential, ER may serve as an excellent diagnostic tool by enabling accurate histopathological staging of the tissue specimen on which further patient management is based. In the esophagus, the most commonly used resection methods are endoscopic mucosal resection (EMR) and endoscopic submucosal dissection (ESD) 4 .

Despite both resection techniques having been proven safe and effective for early esophageal neoplasia 5 6 7 8 , patients with esophageal varices are considered a high-risk group owing to an increased risk of massive bleeding 9 . However, most of these patients are also precluded from major esophageal surgery because of portal hypertension resulting in collateral blood vessels 10 . In addition, a diagnosis of esophageal neoplasia may exclude selected patients from liver transplantation 11 . Therefore, endoscopic therapy may be the only treatment option for this specific patient category, provided that the prognosis of the underlying liver disease justifies treatment of the early esophageal neoplasia.

Thus far, several approaches have been described in the literature to reduce the bleeding risk and thereby enable ER in these patients. Ideally, varices are eradicated before the planned ER through endoscopic band ligation (EBL), endoscopic injection sclerotherapy (EIS), or transjugular intrahepatic portosystemic shunt (TIPS) 12 13 14 15 . Preventative EBL and EIS, however, is not possible if the neoplastic lesion is located at the esophagogastric junction. In addition to the treatment of varices prior to ER, bleeding may also be prevented by periprocedural interventions, such as direct coagulation 16 .

Although the currently available literature may support the idea that the presence of esophageal varices is not an absolute contraindication to endoscopic management of early esophageal neoplasia, these studies are limited by a small sample size and/or short follow-up. Moreover, many clinicians still regard esophageal varices as a contraindication to endoscopic therapy and thereby withhold patients from referral to a highly specialized center for consideration of endoscopic therapy. In addition, there is no clear overview of all possible preventative measures to mitigate the risk of bleeding. Therefore, we performed a systematic review to evaluate the effectiveness and safety of ER for early esophageal neoplasia in patients with esophageal varices, and to assess the various prophylactic measures taken in these patients. Furthermore, based on the results from the literature and our own clinical experiences, we have formulated several practical recommendations to enable endoscopic treatment in this specific patient category.

## Methods

### Search strategy

A literature search was conducted in Cochrane Library, Embase, and Medline on August 20, 2024. The search strategy consisted of key terms and standardized index terms related to esophageal varices and ER. No restrictions on publication language or publication date were used. The full search algorithm is provided in **Table 1s** in the online-only Supplementary material. Reference lists of all included studies were checked manually to identify additional relevant articles.

### Study selection

After removal of duplicates, titles and abstracts were screened for eligibility by two independent authors (C.N.F. and L.S.B.). Any discrepancies regarding study inclusion were resolved through a consensus meeting. Thereafter, full-text review was independently conducted by the same authors using the following predefined inclusion criteria: (1) studies including patients with esophageal varices and concurrent early esophageal neoplasia, defined as high grade dysplasia or T1 cancer, and (2) studies reporting on the clinical outcomes of EMR and/or ESD. Studies were included irrespective of design or follow-up time. Studies were excluded if they were (1) studies performed in the pediatric population (age <18 years), and (2) review articles, comments, or studies reported as conference abstracts only. Studies that partly met the inclusion criteria were discussed individually with a third assessor (B.L.A.M.W.) to resolve any disagreement on final inclusion.

As an addition to the available studies found through the literature search, we have included unpublished data from an observational cohort, consisting of patients treated between January 2014 and December 2023 at three Dutch centers with a tertiary referral function for endoscopic management of early esophageal neoplasia. Please refer to **Text 1s** for further details on this additional retrospective cohort.

### Data extraction

Data from all included studies were extracted independently by two members of the research team (C.N.F. and L.S.B.). All relevant end points were collected on a standardized data collection form designed prior to data extraction. For each study, the following variables were collected: first author, year of publication, country of origin, study design, sample size, patient characteristics including age and Child–Pugh score, size of varices, ER method, worst histopathology of ER specimen, and adverse event rate. In addition, data on prophylactic measures to prevent bleeding were collected.

### Quality assessment

The methodological quality of the studies was evaluated using the Joanna Briggs Institute critical appraisal tools for case series and case reports ( **Table 2s** ) 17 18 . This tool consists of 10 and 8 criteria for case series and case reports, respectively. Eligible cohort studies were assessed using the appraisal tool for case series. The quality of case series or cohort studies was classified as good when ≥9 criteria were met, medium when 6–8 criteria were met, and poor quality when ≤5 criteria were met. Similarly, case reports were classified as good, medium, or poor when meeting ≥7, 5–6, or ≤4 criteria, respectively.

### Study end points

Based on the definitions shown in **Table 3s** , we defined several outcomes. The study end points regarding effectiveness included rates of (1) en bloc resection, (2) radical resection, and (3) curative resection. The study end points regarding safety included (1) the incidence of prophylactic measures to prevent variceal bleeding, (2) the adverse event rate, including periprocedural or delayed bleeding, perforation, and esophageal stricture requiring endoscopic dilation, and (3) procedure-related mortality.

### Ethics

This systematic review was conducted in accordance with the Preferred Reporting Items for Systematic Review and Meta-Analyses (PRISMA) guidelines ( **Table 4s** ) 19 , and was registered in the international prospective register of systematic reviews (PROSPERO CRD42024529958).

### Statistical analysis

No statistical analysis was performed owing to the considerable heterogeneity of the available literature. Instead, the results from the literature are presented in a descriptive manner, using proportions to report categorical variables and medians with minimum and maximum values (min–max) to describe the distribution of continuous data.

## Results

### Search results

**Fig. 1:**
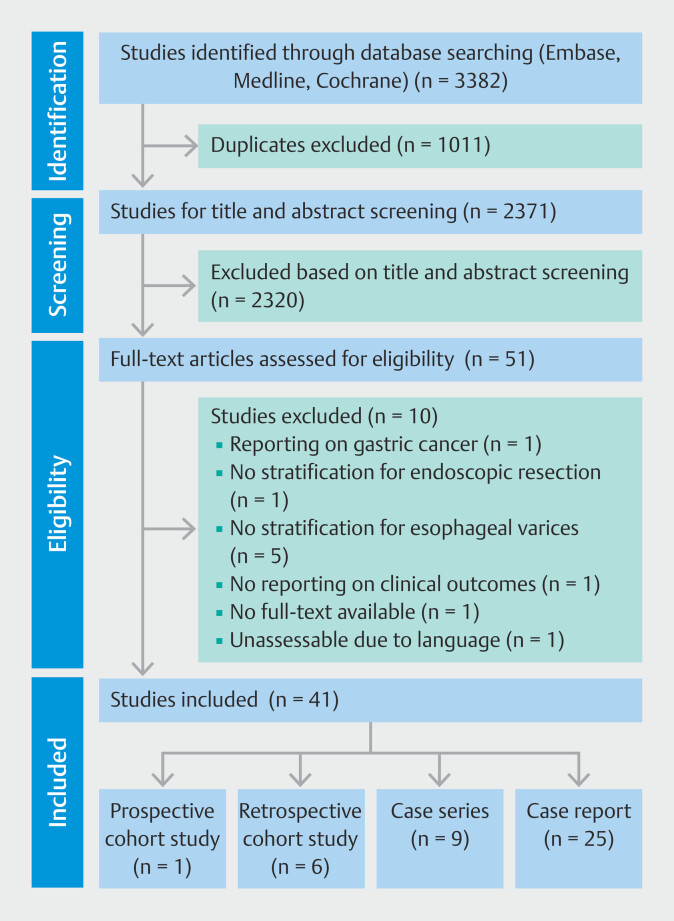
Flow diagram depicting study selection process through a literature search in accordance with the Preferred Reporting Items for Systematic Review and Meta-Analyses guidelines 19 . In addition to the six retrospective cohort studies retrieved through the literature search, our own unpublished cohort was also included in the analysis (supplementary material, Text 1).

The initial search yielded 3382 records, of which 2371 records were screened for title and abstract after removal of duplicates. A total of 51 potentially relevant publications were retrieved for full-text analysis. After assessment for eligibility, 10 publications were excluded based on the inclusion and exclusion criteria 20 21 22 23 24 25 26 27 28 29 . Finally, 41 publications were included ( Fig. 1 ) 9 12 13 14 15 16 30 31 32 33 34 35 36 37 38 39 40 41 42 43 44 45 46 47 48 49 50 51 52 53 54 55 56 57 58 59 60 61 62 63 64 . Reference lists of all included studies were manually checked, but did not identify additional eligible articles. In addition to the available studies found through the literature search, the analysis hereafter also includes unpublished retrospective data from our own clinical practice ( **Text 1s** ).

### Study characteristics and quality assessment

The included publications consisted of 1 prospective cohort study, 7 retrospective cohort studies (including our own unpublished cohort), nine case series, and 25 case reports ( Fig. 1 ). The methodological quality of the case reports was mostly evaluated as good (17/25; 68%), although eight case reports (8/25; 32%) were scored as medium quality ( **Table 5s** ). While all included cohort studies (8/8; 100%) were assessed to be of good quality, the quality of the cases series varied widely, with three publications (3/9; 33%) evaluated as good, five articles (5/9; 56%) as medium, and one case series as poor (1/9; 11%) ( **Table 6s** ). The studies included a total of 186 patients, with individual sample sizes ranging between 1 and 30 patients ( **Table 7s** ). The Child–Pugh score was reported inconsistently, especially in the case reports. Esophageal varices were predominantly classified as small, defined as a size of <5mm (128/186; 69%).

### Prophylactic measures

**Fig. 2:**
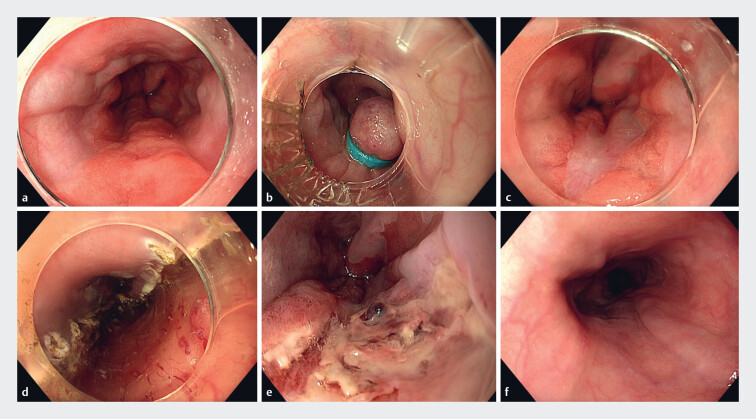
Endoscopic resection after eradication of esophageal varices. **a** Endoscopic images of a patient with a C1M5 Barrett esophagus containing a Paris 0-IIa lesion of 30 mm covering a third of the esophageal circumference. **b** As a result of post-alcoholic liver cirrhosis classified as Child–Pugh A, small esophageal varices were detected and simultaneously treated with endoscopic band ligation (EBL). **c** EBL was repeated after 2 weeks, during which multiple scars of the previous EBL session were seen. **d** Two weeks later, endoscopic submucosal dissection under octreotide administration was technically successful, with histopathology revealing a moderately differentiated T1sm1 adenocarcinoma with no lymphovascular invasion, but a tumor-positive lateral resection margin. **e** The patient returned 1 week later with delayed bleeding due to an ulcer with a visible vessel at the resection site, which was treated with an adrenaline injection and coagulation. **f** Three months later, the resection scar was completely regenerated with squamous epithelium, without any signs of local recurrence. The patient then entered strict endoscopic follow-up considering his comorbidities.

**Fig. 3:**
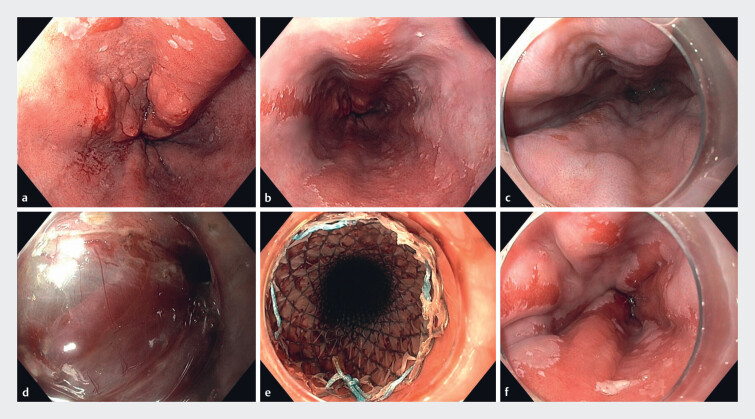
Endoscopic resection in the presence of esophageal varices. **a** Endoscopic images of a patient with a C3M7 Barrett esophagus containing a Paris 0-Is/IIa lesion of 30 mm covering half of the esophageal circumference. **b** During the same endoscopy, large esophageal varices were seen as a result of post-alcoholic liver cirrhosis classified as Child–Pugh A. **c** Despite successful placement of a transjugular intrahepatic portosystemic shunt, varices were still present during the endoscopic submucosal dissection (ESD) procedure 3 months later. **d** During dissection under octreotide administration, multiple collateral vessels were encountered and prophylactically coagulated using the ESD knife and a hemostatic forceps. **e** As an additional safety precaution, a fully covered metal stent was temporarily placed to prevent delayed variceal bleeding. **f** Three months after the radical and curative resection of a moderately differentiated T1m3 adenocarcinoma with no lymphovascular invasion, the resection scar was almost completely regenerated with squamous epithelium, without any signs of local recurrence.

As shown in **Table 7s** , the most widely adopted prophylactic measure was the eradication of varices prior to ER through EBL in 72/186 patients (39%) ( Fig. 2 ). Despite prior EBL, direct varix coagulation was still necessary in 12/72 patients (17%) as a result of persistent varices. Other frequently described prophylactic measures included EIS (22/186; 12%), direct varix coagulation (18/186; 10%), TIPS (9/186; 5%), and a combination of EBL and EIS (5/186; 3%). The ligate-and-let-fall-off approach, typically used when the lesion was located on top of a varix, was adopted in 12/186 patients (6%), preceded by EBL in three (3/12; 25%) of these cases. With this strategy, the lesion and underlying varix are ligated using rubber bands while subsequent snaring is omitted 12 . Eventually, the lesion will become necrotic and fall off spontaneously. In two patients (1%), a temporary metal stent was placed after submucosal varices started to bleed during the resection ( Fig. 3 ). In the remaining 46/186 patients (25%), no preventative measures were described.

### ER outcomes

While 34/186 patients (18%) underwent EMR for esophageal neoplasia, the majority of patients were treated with ESD (152/186; 82%) ( **Table 7s** ). As shown in **Table 8s** , most patients were diagnosed with early squamous cell neoplasia (130/186; 70%).

In all studies, no procedure-related mortality was observed. Adverse events were seen in 27/186 patients (14%) with one patient experiencing two separate complications ( **Table 7s** ). The most common adverse event was periprocedural bleeding (12/186; 6%), followed by esophageal stricture requiring dilation (5/186; 3%), delayed bleeding (5/186; 3%), infection (4/186; 2%), and perforation (2/186; 1%). Periprocedural bleeding was resolved endoscopically in nine cases 51 58 60 62 64 , but resulted in a prematurely terminated (n = 2) or macroscopically incomplete (n = 1) resection in three patients 9 54 61 . In the five patients with delayed bleeding, the hemorrhage was resolved without (n = 2) or with (n = 3) endoscopic intervention 57 58 63 .

### Histopathological outcomes

The histopathological characteristics of the ER specimens were reported inconsistently throughout the included studies ( **Table 8s** ). Based on the available data, the radical and curative resection rates were 137/160 (86%) and 59/82 (72%), respectively.

## Discussion

Although the presence of esophageal varices is still considered by many endoscopists to be an absolute contraindication to endoscopic therapy for early esophageal neoplasia, the currently available literature evaluated in our systematic review demonstrates that ER is safe and effective in this subset of patients, as reflected by the relatively low adverse event rate and substantial curative resection rate. These favorable outcomes of ER highlight that patients with early stage cirrhosis or noncirrhotic portal hypertension should be considered for endoscopic therapy. Naturally, the risks of ER should be balanced against the severity of comorbidities and the risk of dying from causes other than esophageal cancer, including the complications of portal hypertension.

Unfortunately, the studies included in our review were inconsistent in their reporting of the histopathological outcomes after ER. Nevertheless, the curative resection rate of 72% derived from the available data is in line with the curative resection rates in the general Western population, which range from 72% to 96% for Barrett esophagus-related neoplasia, and from 46% to 74% for squamous cell neoplasia 5 65 66 67 68 , demonstrating the effectiveness of ER in patients with esophageal varices. The results from our systematic review are also comparable to those from other large studies on patients with liver cirrhosis or portal hypertension, which demonstrate a curative resection rate of 63%–76% 21 22 .

Regarding safety, the available literature reported a combined periprocedural and delayed bleeding risk of 9%. This incidence is slightly higher when compared with the bleeding risk after ER in the general population, which varies from 2% to 5% 6 7 69 70 . Nonetheless, the majority of bleedings could be managed endoscopically, except for three periprocedural bleedings, which resulted in an incomplete resection. When combining the data from our systematic review, this is a mere 2% (3/186) of all ER procedures. More importantly, the common message in all studies is that none of the patients suffered life-threatening bleeding or death related to the ER procedure.

As a direct consequence of the low number of cases available in the literature, we included all studies irrespective of ER technique. Even though this may have introduced a certain level of heterogeneity, both EMR and ESD are currently advised for different types of esophageal lesions and may therefore be complementary to each other 1 2 . Nevertheless, the present literature on patients with esophageal varices suggests that ESD should be preferred over EMR. In our systematic review, only two cases of delayed bleeding were observed after ESD (2/152; 1%), whereas EMR seemed to carry a higher periprocedural and delayed bleeding risk (9% [3/34] and 3% [1/34], respectively). This observed difference is in line with a recent, large observational study on patients with liver cirrhosis or portal hypertension, which reported a lower bleeding risk after ESD 21 , but these results should be interpreted with caution given the limited number of patients treated with EMR in both studies. Nevertheless, ESD holds the practical advantage that any visible perforating varices can be directly coagulated during resection.

**Fig. 4:**
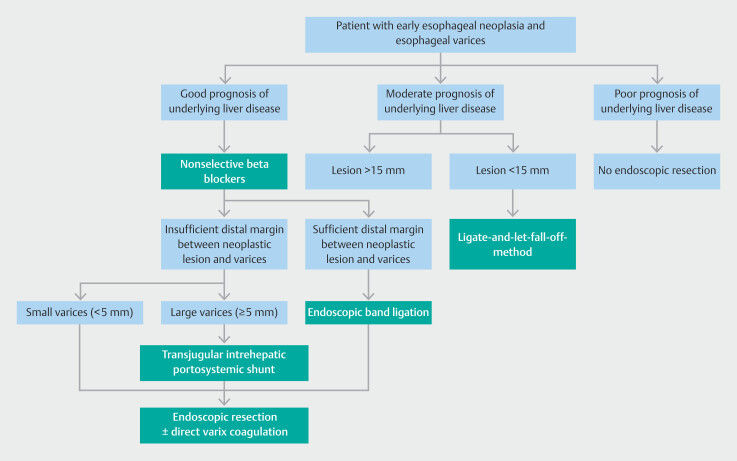
Flow chart to help guide clinical decision making on prophylactic measures to mitigate the bleeding risk of endoscopic resection (ER) in patients with concurrent early esophageal neoplasia and esophageal varices. This flow chart is based on the limited available literature as well as expert opinion. Besides the possible prophylactic measures indicated in green, administration of a somatostatin analog during ER should be considered to reduce the splanchnic blood flow.

An important requirement to enable ER in this specific patient category is the application of adequate prophylactic measures to prevent variceal bleeding. All studies provide a variety of possibilities, making it hard to choose which prophylactic measure is most suitable for each individual patient. Therefore, based on the available data and expert opinion, we present a flow chart with a number of clinical scenarios to help guide clinical decision making ( Fig. 4 ). Primarily, irrespective of the size of the varices, we would advocate reducing the splanchnic blood flow by initiating nonselective beta blockers prior to ER, assuming no contraindications exist. In addition, we advise administration of a somatostatin analog at the start of the ER procedure for at least 3 days, along with prophylactic antibiotics to prevent peritonitis. Besides these drug options, we believe that repeated EBL sessions should generally be the preferred method to completely eradicate the varices prior to the ER procedure, under the condition that there is enough space distally from the neoplastic lesion to ligate the varices. Otherwise, TIPS can be considered in patients with large varices, while periprocedural coagulation of visible varices remains an option in all cases. Although EIS is described in several case reports as a possible preventative measure, we believe that the risk of inducing fibrosis and thereby potentially complicating a re-resection is too high, especially considering the adequate alternatives. In addition to the flow chart, we have formulated several practical recommendations that may be helpful for endoscopists who perform ESD in the presence of esophageal varices ( Table 1 ).

**Table:** **Table 1** Practical recommendations for endoscopic submucosal dissection in the presence of esophageal varices, with the aim of preventing variceal bleeding. These practical recommendations are based on expert opinion derived from our own clinical experience.

Procedure step	Tips and tricks
Mucosal lifting	Start ejecting the lifting fluid just before the injection needle punctures the mucosa, preferably using a colloid solution to decrease the blood flow in the varices by counter pressure.
	Inject a large volume of lifting fluid directly adjacent to the varices to ensure adequate lifting of the mucosa above the varix, and to avoid cutting through a varix during the mucosal incision.
Mucosal incision	Make a careful but *complete* mucosal incision outside the direct range of the varices to expose the varices but prevent variceal bleeding at the same time.
	Prophylactically coagulate and cut every exposed varix with either the endoscopic submucosal dissection knife or a hemostatic forceps before proceeding to the submucosal dissection.
Submucosal dissection	Dissect deeply through the submucosa by staying close to the muscular layer with the aim of avoiding the varices by dissecting underneath them.

To the best of our knowledge, this is the first systematic review to evaluate the safety and effectiveness of ER in patients with esophageal varices, and provides a complete and thorough overview of the available literature on this subject according to PRISMA guidelines 19 . Additionally, our synopsis of possible measures to prevent variceal hemorrhage enabled us to formulate tips and tricks to help guide clinical decision making. Another strength is the addition of our own unpublished observational cohort, for which data were collected by a dedicated research fellow to ensure high quality. The expert setting of this cohort may have contributed to the observed low adverse event rate, endorsing the need to treat these challenging cases only in dedicated centers with the necessary expertise.

This study also has several limitations that need to be acknowledged. The literature search did not allow for a meta-analysis owing to the substantial heterogeneity of the available studies, which mainly consisted of case reports and case series. The between-study heterogeneity was partly the result of differences in ER techniques, histopathological subtypes, applied prophylactic measures to mitigate bleeding, study populations, and outcome parameters. In addition, the retrospective nature of most included studies holds an inevitable risk of selection bias. As ER might have been omitted in some patients due to the presence of varices or expected poor prognosis, the currently available literature may possibly include a more favorable subset of patients. Another limitation is the lack of an international, standardized protocol for reducing the risk of variceal bleeding, resulting in a wide variety of prophylactic measures initiated. On the other hand, this also highlights the multimodal character of the “toolbox” available for facilitation of endoscopic therapy in this challenging subset of patients. Regarding prophylactic measures, it should also be mentioned that the periprocedural bleeding risk may have been underestimated as we considered the use of direct varix coagulation as a preventative measure when, in reality, this option may have also been applied to stop severe variceal bleeding. Finally, apart from four publications that included more than 20 patients, most studies had a small sample size, which is a direct consequence of the low prevalence of this specific patient category.

To conclude, ER appears to be a safe and effective option in selected patients with concurrent early esophageal neoplasia and esophageal varices, provided that adequate prophylactic measures to prevent bleeding are tailored to the patient and applied by an experienced endoscopist. Therefore, we strongly recommend referring patients with concurrent early esophageal neoplasia and varices to a highly specialized center for consideration of endoscopic therapy.

## Supplementary Material

Supplementary Material Zusatzmaterial
