# Supplementary material for: Endoscopic resection of early esophageal neoplasia in patients with esophageal varices: a systematic review
Source: Endoscopy. 2025 Feb 24;57(7):769–77. doi: 10.1055/a-2524-4148 (PMC12224665; doi:10.1055/a-2524-4148)
Supplement: Supplementary file 1 — Supplementary Material [file 10-1055-a-2524-4148_25451650.pdf]

Frederiks CN, Boer LS, Gloudemans B, Alvarez Herrero L, Bergman JJGHM, Pouw RE, Weusten BLAM.

PREVENTION OF BLEEDING AFTER ENDOSCOPIC RESECTION OF EARLY ESOPPHAGEAL NEOPLASIA IN PATIENTS WITH ESOPHAGEAL VARICES: A SYSTEMATIC REVIEW

Text 1s. Details of unpublished retrospective observational cohort.

Methods

Patient selection

For this retrospective, observational cohort, patients were included at three Dutch centers with a tertiary referral function for endoscopic management of early esophageal neoplasia. Patients were eligible for study participation if they were ≥18 years of age, had a lesion in the esophagus suspected for early neoplasia (i.e. high-grade dysplasia or T1 cancer) with an indication for EMR or ESD, had esophageal varices prior to or at the time of ER, and were treated between January 2014 and December 2023.

Endoscopic resection procedure

All procedures were performed by dedicated endoscopists with extensive experience in ER in the upper gastrointestinal tract (L.A.H., J.B., R.P., and B.W.). Patients first underwent a high-definition imaging endoscopy to evaluate whether the early neoplastic lesion was amenable for ER including documentation of size, circumferential extent, Paris classification and proximity to varices. Based on these endoscopic characteristics, the endoscopist could opt for an EMR or ESD during a subsequent procedure. EMR was performed using the Captivator (Boston, Marlborough, MA, USA) or Duette device (Cook, Bloomington, IN, USA), while ESD was performed using the DualKnife (Olympus, Tokyo, Japan), ITKnife nano (Olympus, Tokyo, Japan), or Splash M-Knife (PENTAX, Tokyo, Japan). When the lesion was located on top of a varix, the endoscopist could also use the ligate-and-let-fall-off approach. Follow-up after ER was performed in accordance with local standard of care, taking into account treatment outcome and patient’s comorbidities.

Endoscopy | DOI 10.1055/a-2524-4148 | © The Author(s).

Histopathological evaluation

All ER specimens were processed according to routine clinical care after pinning on paraffin and fixation in formalin. Histopathological evaluation of these specimens was performed by the local expert gastrointestinal pathologist of each participating site.

Prophylactic measures

Since there was no standardized protocol, prophylactic measures to reduce the risk of variceal hemorrhage were initiated at the discretion of the endoscopist. Besides the use of beta blockers to reduce splanchnic blood flow, a somatostatin analogue could be intravenously administered around the ER procedure. EBL was an option either prior to or during ER in cases of varices distal to the neoplastic lesion. Another prophylactic alternative considered in carefully selected patients was TIPS placement. This prophylactic measure was performed prior to the ER procedure in a specialized tertiary center, after which patency of the stent and reduction in hepatic venous pressure gradient was checked. During the ER procedure, direct coagulation using the ESD knife or the Coagrasper hemostatic forceps (Olympus, Tokyo, Japan) provided an option to prophylactically treat visible varices or resolve small periprocedural bleedings. Lastly, an esophageal stent could be temporarily placed after ER to prevent delayed bleeding.

Study endpoints

The outcomes for this observational cohort were comparable to the study endpoints defined for the systematic review (Supplementary Table 3). As study endpoints regarding effectiveness, we observed the rates of (1) en bloc resection; (2) radical resection; and (3) curative resection. The study endpoints regarding safety included (1) the incidence of prophylactic measures to prevent variceal bleeding; (2) the adverse event rate including periprocedural or delayed bleeding, perforation, and esophageal stricture requiring endoscopic dilation; and (3) procedure-related mortality. We additionally assessed (1) the recurrence rate, defined as the percentage of patients with recurrent neoplasia within 1cm of the original ER scar, lymph node metastases, and/or distant metastases during endoscopic follow-up; and (2) overall survival, defined as the time from ER to the date of death from any cause or the date of last follow-up.

Data collection and management

Patients were identified by systematically screening endoscopy databases at each participating site. All relevant data regarding baseline characteristics, imaging, treatment and follow-up were extracted from the electronic patient files into a standardized, online database by a dedicated research fellow (MD). Varices were divided according to the Baveno VII classification into isolated esophageal,

Endoscopy | DOI 10.1055/a-2524-4148 | © The Author(s).

gastroesophageal type 1 (i.e. extending along the lesser curvature of the stomach) or gastroesophageal type 2 (i.e. extending along the greater curvature of the stomach) with the size defined as either small (<5 mm) or large (≥5 mm). Data concerning development of recurrences as well as date and cause of death were checked with general practitioners and referral hospitals. All fields were examined for missing data, remarkable values or outliers with completion or correction where possible.

Statistical analysis

For descriptive statistics, mean and standard deviation (SD) or median with 25th and 75th percentiles (25p-75p) were used for data with a normal or skewed distribution, respectively. Categorical variables were reported as proportions. Outcome variables were reported with 95% confidence intervals (CIs) obtained using simple bootstrapping with 1,000 samples. Kaplan-Meier plots were used for data on overall and recurrence-free survival, with median time to event presented with 95% CIs. Patients were censored upon termination of the study (i.e. 31 December 2023) if follow-up was still ongoing, or upon date of death or last available follow-up if patients were lost to follow-up. Statistical analysis was performed with Statistical Software Package SPSS version 27 for Windows (IBM Corp., Armonk, NY, USA).

Ethics

The Medical Research Ethics Committees United declared that the study was not subject to the Medical Research Involving Human Subjects Act and waived the need for formal ethical review. Where possible, electronic patient files were checked for registration of objection on participation in research. Otherwise, patients were approached through an opt-out procedure with the possibility to object against participation in the study.

Results

Study population

In this observational cohort, 23 patients were included whom were all male with a median age of 69 years (25p-75p 65-74). Patients were classified as either ASA II (9/23; 39%) or ASA III (14/23; 61%). Esophageal varices were mainly caused by early stage liver cirrhosis (Child Pugh A 18/23 [78%], Child Pugh B 1/23 [4%], Child Pugh C 1/23 [4%], unknown 1/23 [4%]), although a few patients were diagnosed with non-cirrhotic portal hypertension (2/23; 9%). The main cause of liver cirrhosis was alcoholic (12/23; 52%) or metabolic dysfunction associated steatohepatitis (4/23; 18%). In the majority of patients, the varices were classified as isolated esophageal varices (20/23; 87%) and small of size (16/23; 70%).

Endoscopy | DOI 10.1055/a-2524-4148 | © The Author(s).

Prophylactic measures

The most frequently used prophylactic measures to prevent bleeding were octreotide administration (9/23; 39%) and/or direct varix coagulation during resection (15/23; 65%). In the majority of patients with small varices (9/16; 56%), octreotide administration (n = 1), direct varix coagulation (n = 5) or a combination of both measures (n = 3) was the only preventive action taken. EBL prior to ER was applied in three patients (3/23; 13%) of which one case had large varices. In two of these patients the varices were completely eradicated after median 2 EBL sessions (min-max 2-3) (Figure 2), while additional prophylactic ligation during the ER procedure was required in the other patient who had remaining small varices distal from the lesion. A TIPS was placed prior to ER in three patients (3/23; 13%), either due to the large size of the varices (n = 2) or the large extent of the neoplastic lesion located on top of a small varix (n = 1). In one of these patients, TIPS placement was complicated by occlusion of the left hepatic artery resulting in necrosis of the left liver lobe. In another patient, large varices were still present during the ESD procedure despite periprocedural octreotide administration and a well-functioning TIPS placed 11 weeks prior to ESD (Figure 3). To control mild persistent oozing and to prevent larger bleeding, a fully covered stent was additionally placed which could be removed after three days without complications. The ligate-and-let-fall-off approach was adopted in one patient (1/23; 4%), since the 15mm neoplastic lesion was located on top of a large varix at the cardia. In five patients (5/23; 22%), no measures were taken to reduce the bleeding risk. While four of these patients had small varices, the presence of varices was unknown at time of the EMR in the other remaining patient resulting in a periprocedural bleeding upon snaring of the lesion (1/23; 4% [95% CI 0%-13%]). This varix bleeding was eventually resolved after hemoclip placement in combination with octreotide administration.

Endoscopic resection outcomes

The median length of the resected neoplastic lesions was 30mm (25p-75p 15-40) with a median circumferential extent of 25% (25p-75p 20-50). The majority of patients was diagnosed with Barrett esophagus related neoplasia (20/23; 87%), while the remaining cases had esophageal squamous cell neoplasia (3/23; 13%). ESD was the most used resection technique in 17/23 (74%) patients followed by EMR in 5/23 (22%) patients. While one ESD procedure was converted to piecemeal EMR due to inadequate lifting of the neoplastic lesion, a resection specimen was missing in one patient (1/23; 4%) as a result of the ligate-and-let-fall-off method. After a median procedure duration of 85 minutes (25p-75p 59-188), en bloc resection was achieved in 87% (20/23; 95% CI 74%-100%).

While no procedure-related mortality was observed, adverse events were seen in 5/23 (22% [95% CI 4%-39%]) patients. Two patients (2/23; 9% [95% CI 0%-22%]) with small varices

Endoscopy | DOI 10.1055/a-2524-4148 | © The Author(s).

prophylactically treated with EBL prior to EMR (n = 1) or ESD (n =1) experienced delayed bleeding as a result of ulceration at the resection site identified upon endoscopy. While in one patient the bleeding resolved without endoscopic intervention, the other patient was successfully treated for a visible vessel at the ulcus using adrenaline injection and coagulation (Figure 2). Other adverse events included a stricture requiring a total of six endoscopic dilations, a laceration which was prophylactically clipped and an aspiration pneumonia which was treated with oral antibiotics.

Histopathological outcomes

The patient treated with the ligate-and-let-fall-off approach was excluded from this analysis considering a resection specimen was missing. In the remaining patients, early cancer was detected as worst histopathology in the majority of resection specimens (20/22; 91%). The other two resection specimens contained either high-grade dysplasia (1/22; 5%) or non-dysplastic Barrett esophagus (1/22; 5%). Further histopathological assessment revealed a radical resection in 20/22 (91% [95% CI 77%-100%]) patients, while tumor-positive resection margins were found in 2/22 (9%) patients (ESD n = 2). Based on the absence of high-risk features, curative resection was achieved in 16/22 (73% [95% CI 52%-91%]) patients.

Recurrence and survival

During a median follow-up of 34 months (25p-75p 21-52), only one patient (1/23; 4% [95% CI 0%-13%]) experienced a local recurrence two months after a non-curative ESD for a moderately differentiated T1sm3 adenocarcinoma with lymphovascular invasion. This local recurrence was successfully retreated through ESD in combination with EMR. None of the patients developed lymph node or distant metastases. Six (6/23; 26% [95% CI 9%-44%]) patients died during follow-up (median 31 months [25p-75p 13-44] after ER), of which the majority (4/6; 67%) was due to unrelated comorbidities. While no patient died from esophageal cancer, two patients died as a result of complications of liver cirrhosis (variceal bleeding n = 1, end stage cirrhosis n = 1). The 3-year estimated survival probability was 79% (95% CI 60%-100%).

Endoscopy | DOI 10.1055/a-2524-4148 | © The Author(s).

Table 1s Complete search string for literature search conducted on November 1st 2023 in three online databases.

Source	Search strategy
Cochrane	(("Endoscopic Mucosal Resection" OR "Endoscopic Submucosal Dissection" OR "Endoscopic Resection" OR "endoscopic treatment" OR "endoscopic therapy" OR "endoscopic Eradication Therapy") AND ((esophageal NEXT vari*) OR (oesophageal NEXT vari*) OR "varices" OR "varix" OR "portal hypertension" OR "liver cirrhosis" OR "cirrhosis")):ti,ab,kw
Embase	('endoscopic mucosal resection'/de OR 'endoscopic submucosal dissection'/de OR 'endoscopic mucosal resection':ti,ab OR 'endoscopic submucosal dissection':ti,ab OR 'endoscopic resection':ti,ab OR 'endoscopic treatment':ti,ab OR 'endoscopic eradication therapy':ti,ab) AND ('esophagus varices'/exp OR 'portal hypertension'/exp OR 'liver cirrhosis'/exp OR 'esophageal vari*':ti,ab OR varices:ti,ab OR varix:ti,ab OR 'portal hypertension':ti,ab OR 'liver cirrhosis':ti,ab OR 'cirrhosis':ti,ab)
Medline	("endoscopic mucosal resection"[MESH] OR "endoscopic mucosal resection"[tiab] OR "endoscopic submucosal dissection"[tiab] OR "endoscopic resection"[tiab] OR "endoscopic treatment"[tiab] OR "endoscopic therapy"[tiab] OR "endoscopic eradication therapy"[tiab]) AND ("esophageal and gastric varices"[MESH] OR "esophageal vari*"[tiab] OR "oesophageal vari*"[tiab] OR varices[tiab] OR varix[tiab] OR "hypertension, portal"[MESH] OR "portal hypertension"[tiab] OR "liver cirrhosis"[MESH] OR "liver cirrhosis"[tiab] OR cirrhosis[tiab])

Endoscopy | DOI 10.1055/a-2524-4148 | © The Author(s).

Table 2s Joanna Briggs Institute critical appraisal tool for assessing case series and case reports.

**JBI critical appraisal for case series**				
	**Yes**	**No**	**Unclear**	**Not applicable**
1. Were there clear criteria for inclusion in the case series?	☐	☐	☐	☐
2. Was the condition measured in a standard, reliable way for all participants included in the case series?	☐	☐	☐	☐
3. Were valid methods used for identification of the condition for all participants included in the case series?	☐	☐	☐	☐
4. Did the case series have consecutive inclusion of participants?	☐	☐	☐	☐
5. Did the case series have complete inclusion of participants?	☐	☐	☐	☐
6. Was there clear reporting of the demographics of the participants in the study?	☐	☐	☐	☐
7. Was there clear reporting of clinical information of the participants?	☐	☐	☐	☐
8. Were the outcomes or follow up results of cases clearly reported?	☐	☐	☐	☐
9. Was there clear reporting of the presenting sites'/clinics' demographic information?	☐	☐	☐	☐
10. Was statistical analysis appropriate?	☐	☐	☐	☐
**JBI critical appraisal for case reports**				
1. Were patient's demographic characteristics clearly described?	☐	☐	☐	☐
2. Was the patient's history clearly described and presented as a timeline?	☐	☐	☐	☐
3. Was the current clinical condition of the patient on presentation clearly described?	☐	☐	☐	☐
4. Were diagnostic tests or assessment methods and the results clearly described?	☐	☐	☐	☐
5. Was the intervention(s) or treatment procedure(s) clearly described?	☐	☐	☐	☐
6. Was the post-intervention clinical condition clearly described?	☐	☐	☐	☐
7. Were adverse events (harms) or unanticipated events identified and described?	☐	☐	☐	☐
8. Does the case report provide takeaway lessons?	☐	☐	☐	☐
Abbreviations: JBI, Joanna Briggs Institute.				

Endoscopy | DOI 10.1055/a-2524-4148 | © The Author(s).

Table 3s Definitions of study end points.

Outcome parameter	Definition
Delayed bleeding	Hematemesis and/or melena ≥24 hours after endoscopic resection requiring either blood transfusion or endoscopic, radiologic and/or surgical intervention.
En bloc resection	Resection of the target lesion in a single piece.
Radical resection	For ESD, en bloc resection with tumor-free vertical and lateral margins; for EMR, macroscopic radical resection with tumor-free vertical margins.
Curative resection	Radical resection of dysplasia or cancer with histology no more advanced than superficial submucosal cancer (≤T1sm1), well to moderately differentiated without lymphovascular invasion.
Periprocedural bleeding	Variceal bleeding during endoscopic resection requiring either blood transfusion, endoscopic intervention including hemoclip placement, band ligation or stent placement, radiological and/or surgical intervention.
Procedure-related mortality	Mortality due to an adverse event related to endoscopic resection.

Abbreviations: EMR, endoscopic mucosal resection; ESD, endoscopic submucosal dissection.

Endoscopy | DOI 10.1055/a-2524-4148 | © The Author(s).

Table 4s PRISMA 2020 checklist.

Section and Topic	Item #	Checklist item	Page # in submitted ms.
**TITLE**			
Title	1	Identify the report as a systematic review.	Title
**ABSTRACT**			
Abstract	2	See the PRISMA 2020 for Abstracts checklist.	Abstract
**INTRODUCTION**			
Rationale	3	Describe the rationale for the review in the context of existing knowledge.	Introduction p5
Objectives	4	Provide an explicit statement of the objective(s) or question(s) the review addresses.	Introduction p5
**METHODS**			
Eligibility criteria	5	Specify the inclusion and exclusion criteria for the review and how studies were grouped for the syntheses.	Methods p6
Information sources	6	Specify all databases, registers, websites, organisations, reference lists and other sources searched or consulted to identify studies. Specify the date when each source was last searched or consulted.	Methods p6 and Suppl. Table 1
Search strategy	7	Present the full search strategies for all databases, registers and websites, including any filters and limits used.	Methods p6 and Suppl. Table 1
Selection process	8	Specify the methods used to decide whether a study met the inclusion criteria of the review, including how many reviewers screened each record and each report retrieved, whether they worked independently, and if applicable, details of automation tools used in the process.	Methods p6
Data collection process	9	Specify the methods used to collect data from reports, including how many reviewers collected data from each report, whether they worked independently, any processes for obtaining or confirming data from study investigators, and if applicable, details of automation tools used in the process.	Methods p6
Data items	10a	List and define all outcomes for which data were sought. Specify whether all results that were compatible with each outcome domain in each study were sought (e.g. for all measures, time points, analyses), and if not, the methods used to decide which results to collect.	Methods p6 and Suppl. Table 7-8
	10b	List and define all other variables for which data were sought (e.g. participant and intervention characteristics, funding sources). Describe any assumptions made about any missing or unclear information.	Methods p6 and Suppl. Table 7-8

Endoscopy | DOI 10.1055/a-2524-4148 | © The Author(s).

Study risk of bias assessment	11	Specify the methods used to assess risk of bias in the included studies, including details of the tool(s) used, how many reviewers assessed each study and whether they worked independently, and if applicable, details of automation tools used in the process.	Methods p7
Effect measures	12	Specify for each outcome the effect measure(s) (e.g. risk ratio, mean difference) used in the synthesis or presentation of results.	NA
Synthesis methods	13a	Describe the processes used to decide which studies were eligible for each synthesis (e.g. tabulating the study intervention characteristics and comparing against the planned groups for each synthesis (item #5)).	NA
	13b	Describe any methods required to prepare the data for presentation or synthesis, such as handling of missing summary statistics, or data conversions.	NA
	13c	Describe any methods used to tabulate or visually display results of individual studies and syntheses.	Methods p6 and Suppl. Table 7-8
	13d	Describe any methods used to synthesize results and provide a rationale for the choice(s). If meta-analysis was performed, describe the model(s), method(s) to identify the presence and extent of statistical heterogeneity, and software package(s) used.	NA
	13e	Describe any methods used to explore possible causes of heterogeneity among study results (e.g. subgroup analysis, meta-regression).	NA
	13f	Describe any sensitivity analyses conducted to assess robustness of the synthesized results.	NA
Reporting bias assessment	14	Describe any methods used to assess risk of bias due to missing results in a synthesis (arising from reporting biases).	NA
Certainty assessment	15	Describe any methods used to assess certainty (or confidence) in the body of evidence for an outcome.	NA
**RESULTS**			
Study selection	16a	Describe the results of the search and selection process, from the number of records identified in the search to the number of studies included in the review, ideally using a flow diagram.	Results p8 and Figure 1
	16b	Cite studies that might appear to meet the inclusion criteria, but which were excluded, and explain why they were excluded.	Results p8 and Figure 1
Study characteristics	17	Cite each included study and present its characteristics.	Results p8 and Suppl. Table 7-8
Risk of bias in studies	18	Present assessments of risk of bias for each included study.	Results p8, and Suppl. Table 5-6
Results of individual studies	19	For all outcomes, present, for each study: (a) summary statistics for each group (where appropriate) and (b) an effect estimate and its precision (e.g. confidence/credible interval), ideally using structured tables or plots.	Results p8-9
Results of syntheses	20a	For each synthesis, briefly summarise the characteristics and risk of bias among contributing studies.	NA
	20b	Present results of all statistical syntheses conducted. If meta-analysis was done, present for each the summary estimate and its	NA

Endoscopy | DOI 10.1055/a-2524-4148 | © The Author(s).

		precision (e.g. confidence/credible interval) and measures of statistical heterogeneity. If comparing groups, describe the direction of the effect.	
	20c	Present results of all investigations of possible causes of heterogeneity among study results.	NA
	20d	Present results of all sensitivity analyses conducted to assess the robustness of the synthesized results.	NA
Reporting biases	21	Present assessments of risk of bias due to missing results (arising from reporting biases) for each synthesis assessed.	NA
Certainty of evidence	22	Present assessments of certainty (or confidence) in the body of evidence for each outcome assessed.	NA
**DISCUSSION**			
Discussion	23a	Provide a general interpretation of the results in the context of other evidence.	Discussion p10-p12
	23b	Discuss any limitations of the evidence included in the review.	Discussion p10-p12
	23c	Discuss any limitations of the review processes used.	Discussion p10-p12
	23d	Discuss implications of the results for practice, policy, and future research.	Discussion p10-p12
**OTHER INFORMATION**			
Registration and protocol	24a	Provide registration information for the review, including register name and registration number, or state that the review was not registered.	Methods p7
	24b	Indicate where the review protocol can be accessed, or state that a protocol was not prepared.	NA
	24c	Describe and explain any amendments to information provided at registration or in the protocol.	NA
Support	25	Describe sources of financial or non-financial support for the review, and the role of the funders or sponsors in the review.	NA
Competing interests	26	Declare any competing interests of review authors.	Conflicts of interest statement
Availability of data, code and other materials	27	Report which of the following are publicly available and where they can be found: template data collection forms; data extracted from included studies; data used for all analyses; analytic code; any other materials used in the review.	NA

Endoscopy | DOI 10.1055/a-2524-4148 | © The Author(s).

Table 5s Quality assessment of the included case reports based on the Joanna Briggs Institute critical appraisal tools, with overall quality scored as good if ≥7 criteria were met (indicated in *green*), medium if 5–6 criteria were met (*orange*), or poor if ≤4 criteria were met (*red*).

Publication	Description of patient's demographic characteristics	Description of patient's history including timeline	Description of patient's condition at presentation	Description of diagnostic tests including results	Description of therapeutic intervention	Description of patient's condition post-intervention	Identification and description of adverse events	Takeaway lessons provided	Overall quality
Dhaliwal et al, 2020	Yes	Yes	No	Yes	Yes	Yes	Yes	Yes	Good
Endlicher et al, 2004	Yes	Yes	Yes	Yes	Yes	Yes	Yes	No	Good
Hadley et al, 2022	Yes	Yes	No	Yes	Yes	No	No	Yes	Medium
Harada et al, 2009	Yes	Yes	Yes	Yes	Yes	Yes	Yes	Yes	Good
Heo et al, 2022	Yes	Yes	No	Yes	Yes	Yes	Yes	Yes	Good
Hsu et al, 2014	Yes	Yes	No	Yes	Yes	Yes	Yes	No	Medium
Inoue et al, 1991	Yes	Yes	Yes	Yes	Yes	Yes	Yes	Yes	Good
Iwase et al, 2000	Yes	Yes	No	Yes	Yes	Yes	Yes	Yes	Good
Jovani et al, 2015	Yes	Yes	No	Yes	Yes	Yes	No	Yes	Medium
Kikuchi et al, 2023	Yes	Yes	No	Yes	Yes	No	Yes	Yes	Medium
Kolb et al, 2021	Yes	Yes	No	No	Yes	No	Yes	Yes	Medium

Endoscopy | DOI 10.1055/a-2524-4148 | © The Author(s).

Mitsunaga et al, 2017	Yes	No	No	Yes	Yes	No	Yes	Yes	Medium
Mohapatra et al, 2020	Yes	Yes	Yes	Yes	Yes	Yes	Yes	Yes	Good
Nakachi et al, 2022	Yes	Yes	Yes	Yes	Yes	Yes	Yes	Yes	Good
NeSmith et al, 2014	Yes	Yes	Yes	Yes	Yes	Yes	Yes	Yes	Good
Probst et al, 2022	Yes	Yes	Yes	Yes	Yes	Yes	Yes	Yes	Good
Robbins et al, 2022	Yes	Yes	Yes	Yes	Yes	Yes	Yes	Yes	Good
Shiratori et al, 2019	Yes	No	No	Yes	Yes	No	Yes	Yes	Medium
Tian et al, 2023	Yes	Yes	Yes	Yes	Yes	Yes	Yes	Yes	Good
Ueda et al, 2020	Yes	Yes	Yes	Yes	Yes	Yes	Yes	Yes	Good
Ueda et al, 2024	Yes	No	No	No	Yes	Yes	Yes	Yes	Medium
Wang et al, 2019	Yes	Yes	Yes	Yes	Yes	Yes	Yes	Yes	Good
Wang et al, 2022	Yes	Yes	Yes	Yes	Yes	Yes	Yes	Yes	Good
Wong et al, 2024	Yes	Yes	No	Yes	Yes	Yes	Yes	Yes	Good
Zuo et al, 2023	Yes	Yes	Yes	Yes	Yes	Yes	No	Yes	Good

Endoscopy | DOI 10.1055/a-2524-4148 | © The Author(s).

Table 6s Quality assessment of the included case series, prospective cohort studies and retrospective cohort studies based on the Joanna Briggs Institute critical appraisal tools, with overall quality scored as good if ≥9 criteria were met (indicated in *green*), medium if 6–8 criteria were met (*orange*), or poor if ≤5 criteria were met (*red*).

**Publication**	**Clear inclusion criteria**	**Standardized measurement of patients' condition**	**Valid methods for identification of patients' condition**	**Consecutive inclusion of patients**	**Complete inclusion of patients**	**Description of patients' demographics**	**Description of patients' clinical information**	**Description of outcomes or follow-up**	**Description of sites' demographics**	**Appropriate statistical analysis**	**Overall quality**
**Case series (n = 9)**											
Ciocîrlan et al, 2008	Yes	Yes	Yes	Yes	Unclear	Yes	Yes	Yes	No	NA	Medium
Hartgerink et al, 2022	Yes	Yes	Yes	Yes	Yes	Yes	Yes	Yes	Yes	Yes	Good
Künzli et al, 2014	No	Yes	Yes	Unclear	Unclear	Yes	Yes	Yes	No	NA	Poor
Mitsuishi et al, 2013	Yes	Yes	Yes	Unclear	Unclear	Yes	Yes	Yes	No	NA	Medium
Mochimaru et al, 2022	Yes	Yes	Yes	Unclear	Unclear	Yes	Yes	Yes	No	NA	Medium
Prasad et al, 2007	Yes	Yes	Yes	Yes	Unclear	Yes	Yes	Yes	Yes	NA	Medium
Tan et al, 2023	Yes	Yes	Yes	Yes	Yes	Yes	Yes	Yes	Yes	Yes	Good
Sawaguchi et al, 2014	Yes	Yes	Yes	Yes	Yes	Yes	Yes	Yes	Yes	Yes	Good
Uchima et al, 2022	Yes	Yes	Yes	Unclear	Unclear	Yes	Yes	Yes	No	NA	Medium

Endoscopy | DOI 10.1055/a-2524-4148 | © The Author(s).

**Retrospective cohort study (n = 7)**											
Choi et al, 2022	Yes	Yes	Yes	Yes	Yes	Yes	Yes	Yes	Yes	Yes	Good
Mony et al, 2024	Yes	Yes	Yes	Yes	Yes	Yes	Yes	Yes	Yes	Yes	Good
Nakai et al, 2023	Yes	Yes	Yes	Yes	Yes	Yes	Yes	Yes	Yes	Yes	Good
Toya et al, 2023	Yes	Yes	Yes	Yes	Yes	Yes	Yes	Yes	Yes	Yes	Good
Tsou et al, 2016	Yes	Yes	Yes	Yes	Yes	Yes	Yes	Yes	Yes	Yes	Good
Zhang et al, 2023	Yes	Yes	Yes	Yes	Yes	Yes	Yes	Yes	Yes	Yes	Good
Frederiks et al, unpublished data	Yes	Yes	Yes	Yes	Yes	Yes	Yes	Yes	Yes	Yes	Good
**Prospective cohort study (n = 1)**											
Xu et al, 2019	Yes	Yes	Yes	Yes	Yes	Yes	Yes	Yes	Yes	Yes	Good
Abbreviations: NA, not applicable.											

Endoscopy | DOI 10.1055/a-2524-4148 | © The Author(s).

Thieme

Table 7s Overview of the included studies, including patient specific characteristics, prophylactic measures initiated prior or during endoscopic resection, and adverse event rate.

Publication	Country	Patients, n	Age, years[1]	Child-Pugh, n	Size of esophageal varices, n[2]	ER method	Prophylactic measure, n	Adverse event, n
**Case report (n = 25)**								
Dhaliwal et al, 2020	USA	1	65	NR	Small	ESD	Direct coagulation	0
Endlicher et al, 2004	Germany	1	71	B	Small	EMR	EBL	0
Hadley et al, 2022	Lebanon	1	59	NR	Large	EMR	LLFO	NR
Harada et al, 2009	Japan	1	69	B	Large	EMR	EBL + EIS	0
Heo et al, 2022	Korea	1	69	NR	NR	ESD	EBL + direct coagulation	0
Hsu et al, 2014	Taiwan	1	52	NR	Small	ESD	EBL + direct coagulation	0
Inoue et al, 1991	Japan	1	71	B	Large	EMR	EIS	0
Iwase et al, 2000	Japan	1	61	NR	Large	EMR	EIS	0
Jovani et al, 2015	Italy	1	47	NR	Large	ESD	EBL	0
Kikuchi et al, 2023	Japan	1	72	NR	Small	ESD	EIS	0
Kolb et al, 2021	USA	1	62	NR	Large	ESD	Direct coagulation	0
Mitsunaga et al, 2017	Japan	1	80	NR	Small	ESD	Direct coagulation	NR
Mohapatra et al, 2020	USA	1	65	B	Large	ESD	Direct coagulation	0
Nakachi et al, 2022	Japan	1	66	A	Small	ESD	EIS	0
NeSmith et al, 2014	USA	1	66	A	Large	EMR	TIPS	0
Probst et al, 2022	Germany	1	56	A	Large	ESD	TIPS	0
Robbins et al, 2022	USA	1	55	C	Large	ESD	EBL + direct coagulation	0
Shiratori et al, 2019	Japan	1	70	NR	Small	ESD	EBL	0
Tian et al, 2023	China	1	73	NR	Small	ESD	EIS	0
Ueda et al, 2020	Japan	1	67	A	Small	ESD	Direct coagulation	0
Ueda et al, 2024	Japan	1	66	B	Large	ESD	EBL	0

Endoscopy | DOI 10.1055/a-2524-4148 | © The Author(s).

Wang et al, 2019	USA	1	54	NR	Large	ESD	Direct coagulation	0
Wang et al, 2022	China	1	62	A	Small	ESD	EIS	0
Wong et al, 2024	China	1	72	NR	Small	ESD	EBL	0
Zuo et al, 2023	China	1	64	B	Small	ESD	EBL + direct coagulation	NR
**Case series (n = 9)**								
Ciocîrlan et al, 2008	France	4	64 (SD NR)	A or B<br>(n NR)	Small 4	EMR	EBL 3<br>EBL + direct coagulation 1	Periprocedural bleeding 2
Hartgerink et al, 2022	USA	8	61 (10)	NR	Small 4<br>Large 4	EMR	EBL + LLFO 3<br>LLFO 5	0
Künzli et al, 2014	Netherlands	3	66 (50-66)	NR	Small 2<br>Large 1	EMR	EBL 1<br>LLFO 2	0
Mitsuishi et al, 2013	Japan	2	53 (47-59)	A 2	Small 1<br>Large 1	ESD	EIS 2	0
Mochimaru et al, 2022	Japan	2	62 (59-64)	A 2	Small 2	ESD	EBL + EIS 1<br>EBL + direct coagulation 1	Periprocedural bleeding 1
Prasad et al, 2007	USA	4	69 (58-82)	A 2<br>B 2	Small 3<br>Large 1	EMR	EBL 3<br>No measures 1	0
Tan et al, 2023	China	1	52	B	Small	ESD	Stent placement 1	Periprocedural bleeding 1<br>Infection 1
Sawaguchi et al, 2014	Japan	7	69 (55-73)	A 5<br>B 1<br>C 1	Small 6<br>Large 1	ESD	EBL 2<br>No measures 5	0
Uchima et al, 2022	Spain	3	52 (51-63)	A 2<br>B 1	Large 3	EMR	EBL 3	Delayed bleeding 1
**Retrospective cohort study (n = 7)**								
Choi et al, 2022	Korea	8	62 (56-73)	A 5<br>B 2<br>C 1	Small 7<br>Large 1	ESD	No measures 8	Periprocedural bleeding 1<br>Stricture 2
Mony et al, 2024	Canada, China, Japan, USA	23	66 (59-70)	A 9<br>B 13<br>C 1	Small 20<br>Large 3	ESD	EBL 18<br>EIS 1<br>TIPS 3<br>No measures 1	Periprocedural bleeding 1<br>Delayed bleeding 1<br>Perforation 1

Endoscopy | DOI 10.1055/a-2524-4148 | © The Author(s).

Nakai et al, 2023	Japan	27	66 (60-74)	A 17<br>B 10	Small 25<br>Large 2	ESD	EBL 9<br>EIS 1<br>No measures 17	Perforation 1
Toya et al, 2023	Japan	30	70 (56-80)	A 28<br>B 1<br>C 1	Small 18<br>Large 12	ESD	EBL 9<br>EIS 12<br>EBL + EIS 2<br>No measures 7	Periprocedural bleeding 1<br>Stricture 1
Tsou et al, 2016	China	4	58 (46-65)	A 2<br>B 2	Small 3<br>Large 1	ESD	EBL 4	Periprocedural bleeding 2
Zhang et al, 2023	China	7	57 (54-61)	A 4<br>B 3	Small 1<br>Large 6	ESD	Direct coagulation 2<br>EBL 2<br>EBL + direct coagulation 2<br>EBL + EIS + direct coagulation 1	Delayed bleeding 1<br>Infection 2
Frederiks et al, unpublished data	Netherlands	23	69 (65-74)	A 18<br>B 1<br>C 1<br>NR 3	Small 16<br>Large 6	EMR or ESD	Direct coagulation 10<br>EBL 1<br>EBL + direct coagulation 2<br>LLFO 1<br>Octreotide 1<br>TIPS 2<br>TIPS + stent placement 1<br>No measures 5	Periprocedural bleeding 1<br>Delayed bleeding 2<br>Stricture 1<br>Infection 1
**Prospective cohort study (n = 1)**								
Xu et al, 2019	China	5	56 (48-66)	A 2<br>B 2<br>C 1	Small 3<br>Large 2	ESD	EBL + direct coagulation 2<br>TIPS 1<br>No measures 2	Periprocedural bleeding 2<br>Stricture 1

Abbreviations: EBL, endoscopic band ligation; EIS, endoscopic injection sclerotherapy; EMR, endoscopic mucosal resection; ESD, endoscopic submucosal dissection; LLFO, ligate-and-let-fall-off approach; NR, not reported; SD, standard deviation; TIPS, transjugular intrahepatic portosystemic shunt.

[1]Reported as median with minimum and maximum value or mean with standard deviation.

[2]Scored as small in case of size <5mm or classification as F0/F1 or grade 1/2, and as large in case of size ≥5mm or classification as F2/F3 or grade 3. When a description was missing, the size was estimated where possible based on available endoscopic images.

Endoscopy | DOI 10.1055/a-2524-4148 | © The Author(s).

Thieme

Table 8s Overview of the included studies including histopathological characteristics and follow-up.

**Publication**	**Patients**, n	**Worst histopathology**, n	**Invasion depth**, n	**Differentiation grade**, n	**Lymphovascular invasion**, n	**Radical resection**, n	**Curative resection**, n	**Follow up**, months[1]	**Recurrence**, n
**Case report (n = 25)**									
Dhaliwal et al, 2020	1	EAC	Mx	NR	No	Yes	NR	NR	NR
Endlicher et al, 2004	1	SCC	M2	NR	No	Yes	NR	18	Local recurrence 1
Hadley et al, 2022[2]	1	EAC	NA	NA	NA	NA	NA	1	0
Harada et al, 2009	1	SCC	M1	NR	No	Yes	NR	4	0
Heo et al, 2022	1	SCC	SMx	Moderate	Yes	NR	No	18	0
Hsu et al, 2014	1	SCC	Mx	NR	NR	NR	NR	3	0
Inoue et al, 1991	1	SCC	M1	NR	No	Yes	NR	6	0
Iwase et al, 2000	1	SCC	M2	NR	No	NR	NR	12	0
Jovani et al, 2015	1	SCC	SM3	Well	No	Yes	No	NR	NR
Kikuchi et al, 2023	1	SCC	M1	NR	No	Yes	NR	NR	NR
Kolb et al, 2021	1	EAC	Mx	NR	NR	NR	NR	5	0
Mitsunaga et al, 2017	1	SCC	M2	NR	NR	Yes	NR	NR	NR
Mohapatra et al, 2020	1	EAC	M3	Moderate	No	Yes	Yes	NR	NR
Nakachi et al, 2022	1	SCC	M2	NR	No	Yes	NR	60	0
NeSmith et al, 2014	1	HGD	NA	NA	NA	Yes	Yes	15	0
Probst et al, 2022	1	EAC	Mx	Well	NR	Yes	Yes	NR	NR
Robbins et al, 2022	1	EAC	M1	NR	NR	Yes	NR	NR	NR
Shiratori et al, 2019	1	SCC	M3	NR	No	Yes	NR	NR	NR
Tian et al, 2023	1	SCC	SM3	Moderate	NR	Yes	No	4	0
Ueda et al, 2020	1	EAC	M3	NR	No	Yes	NR	2	0
Ueda et al, 2024	1	SCC	M1	NR	NR	Yes	NR	NR	NR
Wang et al, 2019	1	LGD	NA	NA	NA	Yes	Yes	6	0

Endoscopy | DOI 10.1055/a-2524-4148 | © The Author(s).

Wang et al, 2022	1	SCC	SMx	Moderate	NR	Yes	NR	10	0
Wong et al, 2024	1	SCC	NR	Poor	NR	Yes	Yes	NR	0
Zuo et al, 2023	1	SCC	M2	NR	NR	NR	NR	6	0
**Case series (n = 9)**									
Ciocîrlan et al, 2008	4	SCC 4	NR	NR	NR	NR	NR	7 (5-17)	0
Hartgerink et al, 2022[2]	8	NR 3<br>IM 1<br>LGD 1<br>HGD 3	NA	NA	NA	NA	NA	18 (0-84)	0
Künzli et al, 2014[2]	3	EAC 2<br>SCC 1	NA 1<br>Mx 1<br>SM3 1	NA 1<br>NR 2	NA 1<br>NR 2	NA 1<br>NR 1<br>No 1	NA 1<br>NR 1<br>No 1	16 (4-54)	Metachronous lesion 1<br>Local recurrence 1
Mitsuishi et al, 2013	2	SCC 2	M3 2	NR	No 2	Yes 2	NR	25 (20-30)	0
Mochimaru et al, 2022	2	SCC 2	M1 1<br>NR 1	NR	NR	NR 1<br>No 1	NR 1<br>No 1	18 (0-36)	Local recurrence 1
Prasad et al, 2007	4	LGD 1<br>HGD 2<br>EAC 1	NA 3<br>Mx 1	NA 3<br>NR 1	NA 3<br>NR 1	Yes 3<br>No 1	Yes 3<br>No 1	3 (2-4)	Local recurrence 1
Tan et al, 2023	1	SCC	SM2	NR	NR	Yes	No	5	0
Sawaguchi et al, 2014	7	SCC 7	M1 1<br>M2 4<br>M3 2	NR	Yes 1<br>No 6	Yes 6<br>No 1	Yes 6<br>No 1	23 (9-76)	0
Uchima et al, 2022	3	HGD 1<br>EAC 2	NA 1<br>Mx 2	NR	NR	NR	NR	24 (13-36)	0
**Retrospective cohort study (n = 7)**									
Choi et al, 2022	8	SCC 8	NR 6<br>SMx 2	NR	NR	Yes 7<br>No 1	Yes 4<br>No 4	NR	NR
Mony et al, 2024	23	HGD 6<br>SCC 11<br>EAC 6	NA 6<br>Mx 14<br>SMx 3	NA 6<br>Well 8<br>Moderate 6<br>Poor 3	NR	Yes 21<br>No 2	Yes 19<br>No 4	36 (22-55)	Metachronous lesion 1<br>Local recurrence 1

Endoscopy | DOI 10.1055/a-2524-4148 | © The Author(s).

Nakai et al, 2023	27	SIN 2<br>SCC 24<br>EAC 1	NA 2<br>Mx 22<br>SMx 3	NA 2<br>NR 25	NA 2<br>Yes 2<br>No 23	Yes 21<br>No 6	NR 25<br>Yes 2	34 (16-43)	Local recurrence 2
Toya et al, 2023	30	SCC 30	M1 8<br>M2 12<br>M3 4<br>SM1 1<br>SM2 4	NR	NA 1<br>Yes 3<br>No 26	NA 1<br>Yes 24<br>No 5	NR	42 (1-150)	Metachronous lesion 1<br>Local recurrence 1<br>Distant metastasis 1
Tsou et al, 2016	4	SCC 4	Mx 1<br>M3 1<br>SM2 2	NR	NR	Yes 2<br>No 2	NR	23 (6-30)	0
Zhang et al, 2023	7	HGD 5<br>SCC 2	NR	NR	NR	Yes 7	NR	27 (6-51)	Local recurrence 1
Frederiks et al, unpublished data	23	NR 1<br>IM 1<br>HGD 1<br>EAC 17<br>SCC 3	NA 3<br>M3 13<br>SM1 3<br>SM2 2<br>SM3 2	NA 3<br>Good 5<br>Moderate 14<br>Poor 1	NA 3<br>Yes 2<br>No 18	NA 1<br>Yes 20<br>No 2	NA 1<br>Yes 16<br>No 6	34 (21-52)	Local recurrence 1
**Prospective cohort study (n = 1)**									
Xu et al, 2019	5	SCC 5	M1 3<br>M3 1<br>SM1 1	NR	Yes 1<br>No 4	Yes 4<br>No 1	Yes 4<br>No 1	20 (range NR)	0

Abbreviations: EAC, esophageal adenocarcinoma; HGD, high-grade dysplasia; IM, intestinal metaplasia; LGD, low-grade dysplasia; M1, invasion into epithelium; M2, invasion into lamina propria; M3, invasion into muscularis mucosae; Mx, mucosal invasion not further specified; NA, not applicable; NR, not reported; SCC, squamous cell carcinoma; SIN, squamous intraepithelial neoplasia not further specified; SM1, submucosal invasion ≤200µm for SCC or ≤500µm for EAC; SM2, submucosal invasion ≤500µm for SCC or ≤1000µm for EAC; SM3, submucosal invasion >500µm for SCC or >1000µm for EAC; SMx, submucosal invasion not further specified.

[1]Reported as median with minimum and maximum value.

[2]Histopathology was based on the biopsies prior to endoscopic resection when the resection specimen was missing due to the ligate-and-let-fall-off approach.

Endoscopy | DOI 10.1055/a-2524-4148 | © The Author(s).
